# A Review of Modeling Electrical Conductivity in Carbon-Filled Polymer Composites

**DOI:** 10.3390/polym18121461

**Published:** 2026-06-11

**Authors:** Alireza Mohseni, Andrew N. Hrymak

**Affiliations:** Department of Chemical and Biochemical Engineering, The University of Western Ontario, London, ON N6A 5B9, Canada; amohse5@uwo.ca

**Keywords:** electrical conductivity, carbon-filled polymer composites, anisotropic conductivity, filler orientation, percolation theory, conductive-network modeling

## Abstract

Electrically conductive polymer composites (ECPCs) have attracted growing interest in applications requiring lightweight, processable, and electrically functional materials. Their increasing use has created a strong need for reliable models capable of predicting electrical conductivity from component properties, composite composition, and microstructural features. Although classical percolation theory can describe the sharp increase in conductivity near the percolation threshold, it is often insufficient for predicting conductivity over a wider range of filler concentrations or for distinguishing the underlying conduction mechanisms. This review examines the main modeling approaches used for carbon-filled polymer composites, including percolation-centered, homogenization, network-based, and data-driven models. These approaches are compared in terms of their assumptions, required inputs, strengths, and limitations, with emphasis on how they account for filler morphology, orientation, dispersion, tunneling effects, and conductive-network formation. The review also identifies key challenges and future needs, particularly the development of integrated, orientation-sensitive, and physically informed models for predicting anisotropic electrical conductivity in processed ECPCs.

## 1. Introduction

Electrically conductive polymer composites (ECPCs) have attracted interest because they combine the low density and processability of polymers with the electrical conductivity of the conductive fillers, making them appropriate materials for applications such as electromagnetic interference (EMI) shielding [1], electrostatic dissipation (ESD) or antistatic components [2,3], and flexible or piezoresistive sensors for structural and health monitoring [4].

Carbon-based fillers such as carbon black (CB), graphite, graphene nanoplatelets (GNPs), and carbon nanotubes (CNTs), including single-walled carbon nanotubes (SWCNTs) and multi-walled carbon nanotubes (MWCNTs), are widely used in ECPCs because they combine relatively high intrinsic electrical conductivity with distinct particle geometries that strongly influence percolation behavior and charge transport in polymer matrices [5,6]. CNTs have received particular attention because of their one-dimensional structure and very high aspect ratio, often on the order of 10^2^–10^4^ [7], which allows conductive pathways to form at relatively low filler loadings. For example, CNT/polymer composites often percolate at around 1 wt.% and in some cases below 0.1 wt.%, whereas more conventional, lower-aspect-ratio fillers such as carbon black typically require about 3–15 wt.% [3]. This ability to achieve conductivity at relatively low filler loading makes CNTs especially attractive for lightweight conductive composites [6,8]. However, single-filler ECPCs often face practical limitations because carbon nanofillers such as CNTs or graphene-based fillers tend to agglomerate due to strong van der Waals forces between particles [9,10]. This agglomeration makes uniform dispersion more difficult and can increase the percolation threshold and reduce conductivity [6,9,11]. For this reason, hybrid filler strategies have been widely explored. Combinations such as CNT/CB and CNT/graphene have been reported to improve inter-filler connectivity, promote synergistic conductive pathways, and reduce the percolation threshold or enhance electrical conductivity relative to single-filler systems [5,10,12,13].

Key properties of carbon fillers are summarized in Table 1 from the perspective of conductivity modeling. Carbon black consists of nanoscale primary particles that form aggregate structures, so its conductivity contribution is strongly affected by contact resistance, dispersion state, and agglomeration. Graphite and graphene-based fillers have platelet-like morphologies, where lateral size, thickness, orientation, and interparticle contacts influence network formation and anisotropic transport. In contrast, CNTs and carbon fibers are one-dimensional fillers, for which diameter, length, aspect ratio, waviness, and orientation are key descriptors controlling contact probability, percolation threshold, contact resistance, and anisotropic electrical conductivity.

Historically, the electrical behavior of ECPCs has been investigated primarily through experiments, while analytical and numerical models have been developed to interpret observed trends and predict conductivity over a range of filler concentrations [3,21]. A central concept is percolation threshold, which describes the transition from electrically insulating to conductive, where connected electrical pathways form in the polymer matrix [3,22]. This concept is especially important because the percolation threshold marks the onset of conductive-network formation in the polymer matrix and therefore provides a useful basis for predicting the sharp rise in electrical conductivity observed in ECPCs. One of the earliest and most influential approaches connecting percolation to electrical transport was introduced by Kirkpatrick [22], who extended percolation theory to transport problems using random resistor networks and showed how network connectivity governs effective conductance [3].

**Figure 1:**
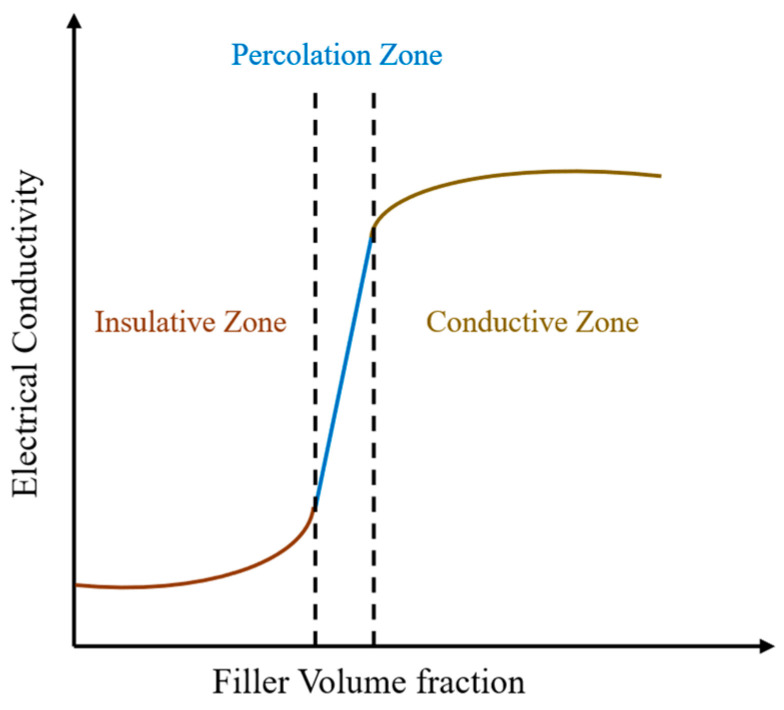
Schematic representation of the typical electrical conductivity–filler loading behavior of ECPCs.

This percolation-based view provides a useful framework for understanding the typical conductivity–filler loading behavior of ECPCs, as schematically illustrated in Figure 1. At low filler loadings, the composite conductivity remains close to that of the insulating polymer matrix because most conductive fillers are isolated. As the filler concentration increases, electron hopping or quantum tunneling across thin polymer gaps, together with direct filler–filler contacts, promotes the formation of conductive pathways. Once a sufficiently connected network forms, conductivity rises sharply near the percolation threshold. The threshold position is governed not only by filler concentration, but also by microstructural factors such as filler morphology, dispersion, orientation, and the tunneling distance between neighboring particles [3,21,23,24,25]. At higher filler contents, the conductivity may reach a nearly constant value; however, this limiting value is often much lower than the intrinsic conductivity of the filler because the overall response is controlled by filler–filler contacts, tunneling resistance, and the topology of the conductive network [3,6,21,26].

In the immediate post-percolation region, the conductivity of ECPCs is often represented by the classical power-law equation shown in Equation (1), where $\sigma_{0}$ is a pre-exponential factor related to filler conductivity, contact resistance, and network structure, $\phi_{c}$ is the percolation threshold, and t is the critical exponent [3]. Although this relation is useful for describing the sharp conductivity increase in this region, the exact definition of $\phi_{c}$ is not always consistent in the literature [27,28]. In addition, the power-law model cannot describe conductivity below the percolation threshold and does not distinguish between different conduction mechanisms [21]. These limitations have motivated the development of broader modeling frameworks for predicting conductivity in ECPCs.

(1) $$\sigma \approx \sigma_{0}{\left(\phi -\phi_{c}\right)}^{t}\;$$

Despite substantial progress, current conductivity models still face important limitations when applied to realistic conductive composites. Many models describe only a limited concentration range, rely on fitted parameters, or represent the microstructure using idealized assumptions such as uniform dispersion and randomly oriented fillers [3,27,29]. In processed composites, however, filler orientation, dispersion, agglomeration, tunneling distance, interfacial effects, and conductive-network formation can vary spatially and interact with one another [30,31,32]. These coupled microstructural features can produce anisotropic electrical conductivity, where the effective conductivity differs between the transverse and through-thickness directions. These limitations highlight the need for modeling frameworks that connect processing-induced morphology with direction-dependent electrical conductivity [13,24,25,33].

Several previous reviews have discussed electrical conductivity in conductive polymer composites, but they have generally emphasized different aspects of the problem. Lux [34] provided an early broad classification of conductivity models for conductive–insulating mixtures, grouping the available approaches into statistical, thermodynamic, geometrical, and structure-oriented models. Bauhofer and Kovacs [6] focused on electrical percolation in CNT/polymer composites and analyzed reported percolation thresholds and critical exponents, showing the wide variability caused by differences in CNT type, dispersion, processing, and composite preparation. Mutiso and Winey [3] reviewed the electrical properties of polymer nanocomposites containing rod-like nanofillers, with particular attention to how filler aspect ratio, dispersion state, interfacial effects, and excluded-volume concepts influence percolation and conductivity. Mohd Radzuan et al. [29] compared several commonly used conductivity models for conductive polymer composites and discussed their applicability across different filler–polymer systems. More recent reviews have focused on narrower topics, including the use of hybrid carbon fillers to improve electrical and piezoresistive performance [5] and the application of Monte Carlo methods to simulate electrical percolation behavior in carbon-filled conductive polymer composites [25]. These studies provide important foundations; however, most of them primarily summarize conductivity behavior, percolation trends, model classes, or specific simulation strategies, rather than critically evaluating how different models can be selected or extended for anisotropic and processing-induced conductivity prediction.

The present review addresses this gap by evaluating electrical-conductivity models from the specific perspective of anisotropic and processing-aware prediction in carbon-filled polymer composites. This distinction is important because, in processed systems, especially injection-molded and compression-molded composites, filler orientation, spatially varying dispersion, agglomeration, tunneling distance, and conductive-network formation are not independent variables; rather, they develop together during processing and can produce different conductivities in the transverse and through-thickness directions [31,32,35]. Orientation-averaged homogenization and analytical models can represent directional effective properties when orientation tensors, probability distribution functions, or orientation distribution functions are available [21,30,36,37], whereas network-based models are more suitable when anisotropy is governed by local connectivity, tunneling distances, contact resistance, and conductive-network topology [38,39,40,41]. Accordingly, the aim of this review is to assess existing electrical-conductivity models for ECPCs with emphasis on their suitability for anisotropic processing prediction. The remainder of the review summarizes the major classes of conductivity models, then discusses the key microstructural factors controlling conductivity, and finally identifies current gaps and future directions for integrated modeling frameworks.

## 2. Electrical Conductivity Models

A wide range of models has been proposed to predict the electrical conductivity of ECPCs, reflecting that conductivity depends on several interacting factors, including filler geometry, orientation, spatial distribution, the intrinsic conductivities of the polymer and conductive particles, interfacial effects, and matrix–particle interactions. Because conductivity models differ in their physical assumptions, input requirements, and ability to represent conductive-network formation, the present review adopts a modeling-approach-based organization. Accordingly, this section is divided into four groups: percolation-centered models, homogenization models, network-based models, and data-driven models. This classification is used not only to describe each model family, but also to evaluate how each approach represents, or fails to represent, direction-dependent transport and processing-induced microstructural features. The discussion therefore emphasizes the range of applicability, required material and microstructural inputs, predictive capability, and main limitations of each model family when applied to different carbon-filled composite systems.

### 2.1. Percolation-Centered Models

Percolation-centered models describe the electrical conductivity of ECPCs based on the formation of a spanning conductive network in the conductivity direction. In these models, the sharp increase in conductivity is associated with a critical filler concentration, known as the percolation threshold, above which connected conductive pathways are formed through the insulating polymer matrix. Because this transition is controlled not only by filler content but also by filler morphology, spatial distribution, and polymer–filler interactions, different percolation-based models have been developed with different levels of physical detail [29,42,43].

#### 2.1.1. Power-Law Equation

The models in this group are mainly valid in the immediate post-percolation region and are used to describe the sharp increase in electrical conductivity that occurs after a connected conductive network begins to form [43]. The classical power-law equation, originally developed in the context of percolation theory, is one of the most common expressions used to describe the sharp increase in electrical conductivity near the percolation threshold and has been widely discussed in reviews and modeling studies of conductive polymer composites [3,26,43]. However, it is only expected to be valid for filler volume fractions slightly above the percolation threshold, and the upper limit of its applicability is still not clearly defined [3].

To use this model as shown in Equation (1), three parameters must be determined: the pre-exponential factor, $\sigma_{0}$, the critical exponent, t, and the percolation threshold, $\phi_{c}$. The pre-exponential factor is affected by filler conductivity, contact resistance, and the structure of the conductive network [3] and it is generally obtained by fitting the model to experimental data. In some studies, such as those by Lux [34] and Clingerman et al. [26], $\sigma_{0}$ was taken to be equal to the electrical conductivity of the filler, which can be considered a simplified form of the more general power-law expression.

The critical exponent, *t*, is commonly related to the dimensionality of the conductive network. In classical percolation theory, *t* is considered a universal transport exponent that describes how the effective conductivity scales with the distance from the percolation threshold. For ideal random resistor networks, *t* is approximately 1.3 in two-dimensional systems and approximately 2.0 in three-dimensional systems [3,26,43]. These values originate from the universality class of lattice or continuum percolation and reflect the dimensionality of the connected conducting cluster rather than the specific chemistry of the composite. However, experimentally estimated values often deviate from these universal predictions [3,26]. Balberg [44] explained that this deviation can occur in tunneling composites because the electrical junctions between neighboring fillers do not all have the same resistance. In these composites, some fillers are in direct contact, while others are separated by thin polymer layers with different interparticle separation distances. Because tunneling resistance increases exponentially with the filler–filler separation distance, small changes in this distance can produce large differences in tunneling resistance. Therefore, the reported critical exponent may reflect not only the formation of a geometrically connected filler network, but also the variation in tunneling resistance along the current-carrying pathways. Consistent with this interpretation, Bauhofer and Kovacs [6] reported non-universal values of t, including values as high as t≈7.5.

The final parameter is the percolation threshold, $\phi_{c}$. In applications of the power-law model, $\phi_{c}$ is often treated as a fitting parameter obtained from conductivity measurements over a range of filler volume fractions. Alternatively, the percolation threshold can be estimated from theoretical percolation analyses of idealized filler networks or from numerical simulations, such as Monte Carlo simulations, that statistically evaluate the formation of a spanning conductive cluster [3,26,38,39,43]. Therefore, the power-law model is most useful in the immediate post-percolation region, where the filler concentration is slightly above $\phi_{c}$ and the conductivity rises sharply. Its main limitations are that it does not describe conductivity below the percolation threshold, is mainly applicable to the immediate post-percolation region, and may lose accuracy at higher filler loadings where conductivity becomes less sensitive to filler concentration or begins to approach a plateau [3,21,27,43]. In addition, its fitted parameters do not explicitly represent mechanisms such as tunneling resistance, contact resistance, filler orientation, dispersion state, or conductive-network morphology [3,21,27,43]. From the perspective of anisotropic conductivity prediction, the main limitation of the power-law model is that it is usually applied as a scalar fitting relation. Unless separate percolation thresholds and critical exponents are fitted for different measurement directions, it cannot directly explain anisotropic conductivity in processed composites.

#### 2.1.2. Mamunya Model

The Mamunya [45] model can be described as a semi-empirical, percolation-based conductivity model developed to represent the conductivity rise in conductive polymer composites above the percolation threshold while also incorporating polymer–filler interaction effects [26,29,43], which are not captured by the classical power-law form. In this model, the conductivity for filler contents above the percolation threshold is written as Equation (2), where $\sigma_{c}$ is the conductivity at the percolation threshold, $\sigma_{m}$ is the conductivity at the maximum packing fraction F representing the upper packing limit in the composite, f is the filler volume fraction, and $f_{c}$ is the percolation threshold. The exponent-like term is not taken as a universal constant but is defined through *k* in Equation (3), with $K=A-B\gamma_{pf}$, so the model explicitly links conductivity evolution to the polymer–filler interfacial tension $\gamma_{pf}$, which is usually estimated from the surface energies of the polymer, $\gamma_{p}$, and the filler, $\gamma_{f}$, using the surface-energy relation in Equation (4) [26,45]. Here, *A* and *B* are empirical constants used to relate *k* to the polymer–filler interfacial tension, and their values are generally obtained from fitting or calibration for a given polymer–filler system. For one-dimensional, high-aspect-ratio fillers such as CNTs, the Mamunya model has also been used together with a maximum packing fraction expression, as shown in Equation (5), where F depends on the filler aspect ratio AR [45]. Therefore, in this formulation, filler geometry is introduced only through aspect ratio, rather than through a detailed description of particle shape.

(2) $$\log\sigma =\log\sigma_{c}+(\log\sigma_{m}-\log\sigma_{c}){\left(\frac{f-f_{c}}{F-f_{c}}\right)}^{k}$$

(3) $$k=\frac{Kf_{c}}{\left(f-f_{c}{)}^{0.75}\right.}$$

(4) $$\gamma_{pf}=\gamma_{p}+\gamma_{f}-2(\gamma_{p}\gamma_{f}{)}^{0.5}\;$$

(5) $$F=\frac{5}{\frac{75}{10+AR}+AR}\;$$

Accordingly, the main assumption behind the Mamunya model is that conductivity above $f_{c}$ is governed not only by filler concentration, but also by interfacial effects such as surface energy [45]. This is the main strength of the model compared with the classical percolation power law, because it adds physically meaningful parameters related to filler geometry and polymer–filler compatibility, rather than relying only on f, $f_{c}$, and a fitted exponent [26,45].

Despite these advantages, the Mamunya model remains semi-empirical and requires material-specific inputs, including surface-energy terms and reference conductivity values at the percolation threshold and maximum packing fraction. Clingerman et al. [26] reported that the model gave the closest agreement with experimental data for several single-filler carbon-filled polymers, including carbon fiber systems, mainly because it incorporated both aspect ratio and surface-energy effects. However, they also showed that its accuracy can decrease for fillers with very high surface energy, such as nickel-coated fibers, and noted that microstructural features such as filler orientation, spatial distribution, agglomeration, and conductive-network morphology are not explicitly represented. Later studies and reviews similarly indicate that the model has been used mainly for single-filler systems and generally performs better above the percolation threshold than below it; for example, Kassim’s [46] carbon black/polymer results agreed well only above the percolation threshold. Therefore, the Mamunya model provides a useful semi-empirical link between percolation theory and interfacial effects, but it remains limited for anisotropic conductivity prediction because anisotropic orientation, spatially varying dispersion, and network connectivity are not directly resolved.

Overall, percolation-centered models are most effective when the main objective is to describe the onset of conductivity, and the sharp conductivity increase near or above the percolation threshold. The classical power-law model is simple and widely applicable for fitting experimental conductivity data in the immediate post-percolation region, but its fitted parameters are often system-specific and may not have unique physical meanings across different filler–polymer combinations. The Mamunya model extends this description by including interfacial and surface-energy-related effects, making it more informative for some single-filler conductive polymer composites above the percolation threshold. However, both approaches remain limited when conductivity is controlled by spatially varying filler orientation, agglomeration, hybrid filler networks, or processing-induced anisotropy. Therefore, percolation-centered models are useful for identifying conductivity transitions and comparing percolation behavior across composite systems, but they are less suitable as standalone predictive tools for direction-dependent conductivity in processed ECPCs.

### 2.2. Homogenization Models

Homogenization models aim to predict the effective electrical conductivity of a heterogeneous polymer composite by replacing its complex microstructure with an equivalent homogeneous medium having the same macroscopic response. In these approaches, the composite is not modeled as an explicit network of individual particles; instead, the influence of the dispersed conductive phase is incorporated through average or mean-field assumptions that relate the effective conductivity to the properties of the matrix and filler, their volume fractions, and, depending on the model, particle shape, aspect ratio, and orientation. Classical examples include the Maxwell–Garnett [47,48] and Bruggeman formulations [43,48,49], while more advanced micromechanics-based approaches such as Eshelby’s equivalent inclusion [50,51] and Mori–Tanaka [52,53] introduce increasingly refined descriptions of inclusion geometry and phase interaction. Because homogenization models replace the heterogeneous composite with an equivalent homogeneous medium, they are primarily intended to predict effective bulk conductivity from constituent properties and averaged morphological descriptors. Their predictive capability may become limited when electrical transport depends strongly on percolating pathways, tunneling events, or explicitly formed conductive networks that are not represented directly in the homogenization framework [42,43].

#### 2.2.1. Maxwell-Garnett Approximation

The Maxwell–Garnett approximation is one of the earliest and most widely used effective-medium models for two-phase composites [43,47,48]. It treats the material as a continuous matrix containing dispersed inclusions, and it was originally developed for spherical inclusions at very low concentrations [43,47]. The model is obtained by replacing each sphere with an equivalent dipole and summing the contribution of all dipoles to determine the effective property of the composite [48]. For this reason, the classical Maxwell–Garnett equation for spherical inclusions can be written as

(6) $$\sigma_{eff}=\sigma_{m}\frac{\sigma_{i}+2\sigma_{m}+2f(\sigma_{i}-\sigma_{m})}{\sigma_{i}+2\sigma_{m}-f(\sigma_{i}-\sigma_{m})}\;$$

where $\sigma_{eff}$ is the effective conductivity, $\sigma_{m}$ is the matrix conductivity, $\sigma_{i}$ is the inclusion conductivity, and f is the inclusion volume fraction. This expression has the same form as the classical Maxwell–Garnett mixing relation originally developed for dielectric permittivity, but it is also commonly applied to effective electrical conductivity by replacing permittivity with conductivity [43,48]. The main assumptions of the model are that the inclusions are isolated, weakly interacting, and embedded in an asymmetric host–inclusion structure, so it is mainly valid in the dilute system [47,48]. Its main strength is its simple analytical form and its clear physical interpretation in terms of constituent properties and filler volume fraction [48]. However, its main limitation is that it becomes inaccurate at higher filler contents, where inclusion–inclusion interactions become important. It also does not describe percolation, because Maxwell–Garnett-type models assume dispersed inclusions in a host medium rather than the formation of a connected conductive network [43,47]. Therefore, the classical Maxwell–Garnett approximation is most appropriate for dilute composites with isolated, weakly interacting inclusions, but it is not sufficient for predicting percolation-driven or anisotropic conductivity in processed ECPCs because filler orientation, spatial dispersion, and directional network formation are not explicitly represented.

#### 2.2.2. Bruggeman Effective Medium Approximation

The Bruggeman model is a classical effective-medium or homogenization approach for estimating the electrical conductivity of two-phase composites and is widely discussed in later effective-medium and percolation-related studies [43,48,49]. In effective-medium models, the real heterogeneous composite is replaced by an equivalent homogeneous material with an effective conductivity, $\sigma_{e}$. The conductivities of the polymer and filler phases are known, while $\sigma_{e}$ is the unknown mixture conductivity to be solved from the model. In contrast to the Maxwell–Garnett model, which treats one phase as inclusions dispersed in a continuous host, the Bruggeman formulation treats the two phases more symmetrically by considering each phase as being embedded in the same equivalent medium with conductivity $\sigma_{e}$ [43,48,49]. Therefore, the model is self-consistent because the surrounding medium used in the calculation is also the effective medium being determined. This makes the Bruggeman model useful at moderate filler concentrations, where the host–inclusion distinction used in dilute Maxwell–Garnett-type models become less appropriate [47,48].

For a two-phase composite composed of isotropic constituents, the classical Bruggeman formulation is commonly written as Equation (7):

(7) $$\phi \frac{\sigma_{1}-\sigma_{e}}{\sigma_{1}+2\sigma_{e}}+(1-\phi )\frac{\sigma_{2}-\sigma_{e}}{\sigma_{2}+2\sigma_{e}}=0$$

where $\sigma_{e}$ is the effective electrical conductivity of the composite, $\sigma_{1}$ and $\sigma_{2}$ are the conductivities of the two constituent phases, and ϕ is the volume fraction of phase 1 [43,48,49]. This implicit relation follows from the self-consistent effective-medium condition, in which the average contribution of the two phases to the macroscopic field is balanced within the equivalent medium [43,47,48,49]. In its classical form, the model was derived from isotropic mixtures and spherical inclusions, and therefore it should be interpreted as a mean-field conductivity relation for statistically homogeneous two-phase systems rather than a direct representation of the actual conductive network inside the composite [43,48,49].

The main advantage of the Bruggeman model is its simple self-consistent form, which makes it attractive for estimating effective conductivity beyond the dilute regime. Nevertheless, it remains a mean-field approximation and does not explicitly describe the detailed geometry of conductive pathways in the composite. As a result, its predictive capability may become limited when charge transport is controlled primarily by percolation-related network formation rather than by average phase behavior. This interpretation is consistent with studies that distinguish effective-medium descriptions from percolation-based treatments and note that effective-medium approximations are mainly applicable away from the percolation threshold, whereas percolation theory becomes necessary as the threshold is approached [43,49]. Although extensions of the Bruggeman model have been proposed for nonspherical inclusions and more complex composite systems, these extensions still represent morphology through simplified descriptors, such as particle shape factors or depolarization factors, rather than through the actual filler positions, tunneling distance, or conductive pathways [47,48]. Therefore, Bruggeman-averages remain useful for estimating averaged effective conductivity, but they are limited when conductivity is governed by percolation, tunneling, or anisotropic network formation [43,48].

#### 2.2.3. Eshelby’s Equivalent Inclusion Method (EIM)

Eshelby’s equivalent inclusion method is a foundational micromechanics-based approach for estimating the effective properties of composite materials and provides the inclusion-level basis for many later mean-field homogenization models, including Mori–Tanaka-type formulations [36,50,52,54]. The method was originally developed for elasticity and is based on the result that, for an ellipsoidal inhomogeneity perfectly bonded to an infinitely extended matrix and subjected to a uniform far-field loading, the field inside the inclusion can be treated as uniform [54,55]. This result makes the method especially attractive because it reduces the local inclusion problem to a tractable analytical form involving a finite set of algebraic relations rather than a full field solution [50]. Later, the same framework was extended to other physical properties, including thermal and electrical conductivity, and has been widely used in conductivity modeling of polymer composites [30,50,51].

The basic idea of EIM is to replace the actual inhomogeneity with an equivalent inclusion embedded in the matrix and to represent the effect of the property mismatch through an internal transformation field [50,54]. The disturbed field inside the equivalent inclusion is related to this transformation field through the Eshelby tensor, which depends mainly on the geometry of the inclusion and the surrounding medium [50,55]. In conductivity problems, this approach allows the local electrical or thermal response of an ellipsoidal particle to be expressed analytically and then incorporated into a homogenization framework for predicting the effective macroscopic conductivity of the composite [30,51].

A representative expression for the inclusion localization tensor in an Eshelby-type conductivity formulation is given by Equation (8) [30,36].

(8) $$A_{f}={\left(I+S\sigma_{m}^{-1}(\sigma_{f}-\sigma_{m})\right)}^{-1}$$

where $A_{f}$ is the inclusion localization tensor, *I* is the identity tensor, *S* is the Eshelby tensor, and $\sigma_{m}$ and $\sigma_{f}$ are the conductivity tensors of the matrix and filler, respectively. This expression shows that the local response of the inclusion depends on the inclusion geometry, represented by *S*, and on the conductivity contrast between the filler and the matrix. For this reason, EIM can be used either as an inclusion-level building block in broader homogenization schemes or as the basis of direct Eshelby-based conductivity models.

The main assumptions of EIM follow directly from this equivalent-inclusion framework. In its classical form, the method assumes that the inclusion can be idealized as ellipsoidal, that it is perfectly bonded to the surrounding matrix, and that the local inclusion problem is embedded in an effectively infinite medium [50,55]. Under these assumptions, EIM provides a convenient analytical link between inclusion geometry, constituent properties, and the resulting local field, which is one of the main reasons why it has been widely adopted in micromechanics-based conductivity modeling [30,50]. However, the classical formulation still represents an averaged inclusion-level description and does not explicitly capture complex microstructural features such as strong filler clustering, irregular particle geometry, imperfect interfaces, or the detailed topology of conductive networks. Therefore, although EIM is a powerful homogenization framework, additional assumptions or extensions are often required when modeling composites in which conductivity is strongly influenced by tunneling, percolation, or pronounced orientation effects [30,50,51].

From the perspective of anisotropic conductivity prediction, Eshelby’s equivalent inclusion method is useful because the inclusion shape and orientation can be represented through the Eshelby tensor, providing a foundation for direction-dependent homogenization. However, by itself, EIM remains an inclusion-level mean-field approach and does not explicitly describe filler–filler contacts, tunneling distances, or the formation of conductive pathways.

#### 2.2.4. Mori-Tanaka Model

Mori–Tanaka-type formulations are among the most widely used micromechanics-based homogenization approaches for predicting the effective properties of composite materials [30,36,53,56]. The method is closely linked to Eshelby’s equivalent inclusion concept, because it uses the Eshelby solution for an ellipsoidal inclusion as the local building block and then introduces a mean-field averaging scheme to account for a distribution of inclusions in the matrix [30,36]. Because the concentration field inside an inclusion can be expressed through Eshelby’s tensor, the Mori–Tanaka scheme provides a practical way to relate constituent properties, inclusion shape, orientation, and volume fraction to the effective macroscopic response of the composite [30,36,56,57].

Using the localization tensor obtained from the Eshelby inclusion problem, a representative Mori–Tanaka estimate of the effective conductivity tensor can be written as Equation (9) [30,36].

(9) $$\sigma_{eff}=\sigma_{m}+\phi_{f}(\sigma_{f}-\sigma_{m})A_{f}{\left[(1-\phi_{f})I+\phi_{f}A_{f}\right]}^{-1}$$

where $\sigma_{eff}$ is the effective conductivity tensor, $\sigma_{m}$ and $\sigma_{f}$ are the conductivity tensors of the matrix and filler, $\phi_{f}$ is the filler volume fraction, and $A_{f}$ is the inclusion localization tensor. This equation shows that the Mori–Tanaka model relates the effective conductivity to constituent conductivities, filler volume fraction, inclusion geometry, and localization effects. In CNT/polymer conductivity modeling, Feng and Jiang [21] used this type of micromechanics framework and extended it by considering electron hopping and conductive-network contributions. Therefore, Mori–Tanaka-type formulations are useful for electrical conductivity modeling when filler shape, volume fraction, interphase effects, and orientation-dependent localization need to be represented in an averaged framework [53,56,57].

Ahmadi and Saxena [30] identified Mori–Tanaka as one of the principal analytical micromechanics theories used to evaluate effective composite properties. They also reviewed several later conductivity-related studies based on Eshelby- and Mori–Tanaka-type formulations. For example, Seidel and Lagoudas [36] used a Mori–Tanaka micromechanics framework to study the electrical conductivity of CNT–polymer nanocomposites, while Feng and Jiang [21] developed a mixed micromechanics model showing that both electron hopping and conductive networks contribute to conductivity.

The main assumptions of the Mori–Tanaka model arise from its mean-field nature. In most conductivity applications, the fillers are idealized as ellipsoidal, cylindrical, or straight rod-like inclusions embedded in a continuous matrix, and each inclusion is assumed to experience an average matrix field rather than the exact local field generated by neighboring fillers. For example, García-Macías et al. [37] assumed straight, uniformly and randomly dispersed CNTs and used an Eshelby–Mori–Tanaka framework in which electron hopping was represented through a conductive interphase around the nanotubes rather than through explicit CNT–CNT tunneling paths. Similarly, Mori–Tanaka-based studies that include orientation effects usually treat the filler orientation statistically, for example, by averaging the response over different orientations rather than resolving the actual spatial arrangement of individual fillers. This makes the model useful for predicting anisotropic effective conductivity, especially when orientation distributions are known, but it also means that local clustering, particle contacts, and detailed network topology are not directly represented.

Another important limitation is related to interfaces and percolation. Classical Mori–Tanaka formulations usually assume perfect bonding or ideal contact between the inclusion and matrix, while real conductive polymer composites may contain interfacial resistance, tunneling distance, imperfect contacts, and polymer-rich interphase regions. Böhm and Nogales [56] showed that interfacial resistance can be introduced into Mori–Tanaka-type schemes, particularly for thermal-conductivity problems, but this requires additional parameters and assumptions. Therefore, although the Mori–Tanaka model is more flexible than simpler effective-medium models, it still cannot naturally predict the formation of a continuous conductive network near the percolation threshold unless it is combined with tunneling, interphase, or percolation-based corrections.

Therefore, Mori–Tanaka-type models are useful for orientation-sensitive homogenization when filler shape and orientation distributions are known, but they remain limited for processed ECPCs in which conductivity is governed by local tunneling distance, CNT–CNT contacts, agglomeration, or percolating network topology.

Overall, homogenization models are useful when the composite can be represented through averaged constituent properties and simplified morphological descriptors. Maxwell–Garnett-type models are most appropriate for dilute systems with isolated inclusions, while Bruggeman-type effective-medium models are more suitable for moderate filler contents where the distinction between matrix and dispersed phase becomes less clear. Eshelby- and Mori–Tanaka-based models provide additional flexibility because they can incorporate inclusion shape, anisotropic filler properties, interphase effects, and orientation distributions. However, their predictive capability can become limited when conductivity is dominated by percolating pathways, tunneling distance, direct filler–filler contacts, or local agglomerated networks, because these features are not explicitly resolved in most mean-field formulations. Therefore, these models are valuable for estimating average or orientation-dependent effective conductivity, but they generally require additional percolation, tunneling, or network-based corrections when applied to highly connected, hybrid, or strongly processing-sensitive carbon-filled composites.

### 2.3. Network-Based Models

Network-based models provide a more explicit framework for predicting electrical conductivity in carbon-filled polymer composites by representing conductive fillers and their interactions as a connected electrical network. In these models, fillers are usually generated inside a representative volume element (RVE) using stochastic or Monte Carlo procedures, and electrical connections are assigned when fillers are in direct contact or separated by a distance small enough for electron tunneling [25,40]. The resulting network is converted into an equivalent resistor network, where intrinsic filler resistance, contact resistance, and tunneling resistance are included and effective conductivity is obtained by solving the electrical network using Kirchhoff’s current law [40,41]. Compared with homogenization models, network-based models can directly account for filler orientation, aspect ratio, spatial distribution, local connectivity, and conductive-path formation [25,41].

In this context, the resistor network model (RNM) can be considered the most common implementation of network-based conductivity modeling. In RNM, each filler segment or filler–filler junction is represented by an electrical resistance, including intrinsic filler resistance, contact resistance, and tunneling resistance through the polymer gap [40,57]. After the conductive network is constructed, a voltage difference is applied across opposite boundaries of the representative volume element, and the effective conductivity is calculated by solving the resulting circuit using Kirchhoff’s current law [41,57].

RNM-based studies differ mainly in how the filler geometry, network connectivity, and resistance components are defined. Early CNT/polymer implementations commonly treated CNTs as randomly distributed straight cylindrical fillers and used a cutoff distance to determine whether neighboring CNTs were electrically connected through direct contact or tunneling [40]. Later studies extended this idea by coupling the electrical network with mechanical deformation, so that CNT positions, orientations, and inter-tube distances could be updated under strain before recalculating the network resistance [38,58]. For example, Alian and Meguid [58] combined Monte Carlo RVE generation, finite element deformation analysis, and modified nodal analysis to predict the piezoresistive response of CNT/epoxy composites, while Chang et al. [38] modeled mechanically deformed three-dimensional conductive networks as equivalent circuits containing intrinsic and tunneling resistances and verified the predictions experimentally for MWCNT/polypropylene composites. These developments show that the RNM is not limited to static conductivity prediction but can also be used to study strain-sensitive conductivity, directional percolation, and processing- or deformation-induced changes in network topology.

A key part of the RNM is the definition of the resistance assigned to each conductive segment or junction. The intrinsic resistance of a filler segment is commonly calculated from its length, cross-sectional area, and intrinsic electrical conductivity, while the resistance between neighboring fillers is often described by contact or tunneling resistance [40,57]. For CNT/polymer composites, tunneling resistance is especially important because two CNTs can contribute to the conductive network even when they are separated by a thin polymer layer. This resistance is commonly estimated using a Simmons-type expression [59]:

$$R_{t}=\frac{h^{2}d}{Ae^{2}\sqrt{2m\lambda }}exp\left(\frac{4\pi d}{h}\sqrt{2m\lambda }\right)$$

where d is the separation distance between neighboring fillers, A is the effective tunneling area, e and m are the electron charge and mass, h is Planck’s constant, and λ is the potential barrier height of the polymer matrix [40,59]. Because $R_{t}$ increases exponentially with the inter-filler distance, small changes in filler spacing, dispersion, or deformation can strongly affect the total network resistance and the predicted composite conductivity [40,60].

A key characteristic of the resistor network model is that it evaluates not only whether a spanning conductive cluster exists, but also how effectively that network carries electrical current. In a simple percolation analysis, a composite is usually considered conductive once a continuous filler cluster connects two opposite boundaries. In RNM, however, the predicted conductivity also depends on the number of conductive pathways, the distribution of current through the network, and the resistance of filler segments and filler–filler junctions [41]. This distinction is important because two microstructures with the same filler volume fraction may both contain a spanning conductive cluster, but still show different conductivities if their number of current-carrying pathways, contact density, or tunneling distances are different [41,57]. For this reason, RNM-based studies have been used to examine the effects of filler length, orientation, curvature, and deformation on the effective electrical conductivity of the composite, meaning the macroscopic conductivity obtained from the overall current response of the simulated RVE, rather than treating the percolation threshold as the only important output [38,61].

Network-based models are particularly useful for anisotropic conductivity prediction because the same microstructure can be solved under electrical loading in different directions, allowing transverse and through-thickness conductivities to be compared directly. However, their practical use is limited by two major sources of uncertainty. The first is microstructural characterization. Accurate network construction requires information about filler position, length distribution, orientation, curvature, dispersion state, agglomeration, and local filler concentration, but these quantities are difficult to measure reliably in three dimensions, especially for CNT- and graphene-based composites. As a result, many network models rely on statistically generated RVEs with assumed filler distributions, which may not fully represent the morphology produced by processing. The second source of uncertainty is the assignment of electrical parameters to network elements. Predicted conductivity can be highly sensitive to the assumed tunneling cutoff distance, tunneling barrier height, effective tunneling area, contact resistance, intrinsic filler resistance, and the rule used to decide whether two fillers are electrically connected [38,39,40,41,57,59,60]. Because several of these parameters are difficult to measure directly and are often fitted or estimated from literature values, different parameter choices can produce different conductivity predictions for similar microstructures. In addition, large RVEs, high filler contents, or highly connected networks can substantially increase the computational cost of solving the resistor network. Thus, network-based models are powerful for representing anisotropic connectivity and conductive networks, but their predictive reliability depends strongly on realistic microstructural inputs, careful parameter selection, and sensitivity analysis.

### 2.4. Data-Driven and Machine Learning Models

Data-driven and machine learning models have recently emerged as complementary approaches for predicting the electrical conductivity of carbon-filled polymer composites. In early applications, artificial neural networks were used to predict the conductivity of CNT/polymer composites from microstructural or material descriptors, showing that nonlinear relationships between filler content, geometry, and conductivity can be learned from numerical or experimental datasets [62,63]. Later studies extended this idea to optimization problems, where neural-network models were combined with design-of-experiment or multi-objective optimization methods to identify composite formulations with improved electrical conductivity [63,64]. More recent work has moved toward data-driven material design [65], interpretable machine learning frameworks [66], models that incorporate percolation and agglomeration effects [67], and graph-based learning methods that explicitly consider conductive-network structure [33]. Therefore, unlike classical percolation, homogenization, and resistor-network models, data-driven approaches do not necessarily begin with a closed-form physical description of electron transport. Instead, they learn input–output relationships from available datasets, which makes them useful for complex systems involving several interacting variables, but also makes their reliability strongly dependent on the quality, size, and physical representativeness of the training data [33,62,66,67].

Early studies such as Matos et al. [62] showed that artificial neural networks (ANN) could be trained to predict the electrical conductivity of CNT/polymer composites from numerical datasets, while later studies applied similar data-driven strategies to experimental datasets, design-of-experiment frameworks, and materials optimization problems [63,64,65,68]. More recent works have further extended this approach by incorporating interpretable machine learning, percolation-based descriptors, and graph-based representations of conductive networks [33,66,67].

In a typical ANN model, the selected input variables are introduced through the input layer, one or more hidden layers are used to capture nonlinear interactions among these variables, and the output layer gives the predicted electrical conductivity. Matos et al. [62] used this approach to predict the electrical conductivity of CNT/polymer composites by training the network on numerical data generated from finite-element simulations. In their work, the ANN acted as a fast prediction model, allowing the conductivity response to be predicted without repeatedly solving the full numerical problem. Razavi et al. [63] applied ANN together with design-of-experiments methods to predict and optimize the electrical conductivity of polymer-based composites, showing that neural networks can be used not only for fitting conductivity data but also for searching favorable material or processing conditions. Similarly, García-Carrillo et al. [64] combined ANN with a multi-objective genetic algorithm to optimize both electrical and thermal conductivity in HDPE/carbon-particle composites. These studies show that ANN models are useful when conductivity depends on several coupled variables and when repeated experimental or numerical evaluations are costly.

More recent studies have extended ANN-based conductivity prediction from simple input–output fitting toward more practical and physically informed frameworks. So et al. [65] used a data-driven approach for the design of electrically conductive nanocomposites, showing how experimental datasets can be used to guide material formulation. Cavalcanti et al. [68] applied ANN models to predict the electrical conductivity of carbon-black-filled polymer composites using variables related to the polymer matrix, filler characteristics, and processing conditions, demonstrating that data-driven models can incorporate practical formulation and manufacturing parameters that are difficult to include in simple analytical equations. Elaskalany and Behdinan [66] further addressed the interpretability problem by combining stochastic multiscale conductivity modeling with interpretable machine learning tools, allowing the relative influence of different input parameters on CNT/polymer conductivity to be evaluated.

A further development in data-driven conductivity modeling is the use of hybrid and network-aware approaches that try to connect machine learning more directly with the physical mechanisms of conduction. Azimi and Sharifzadeh [67], developed a percolation-based model that accounts for nanoparticle aggregation and agglomeration, and combined it with an artificial neural network to predict the electrical conductivity of polymer nanocomposites. Their model incorporated factors such as nanoparticle aggregation, interphase conductivity, tunneling distance, and filler-network formation, showing how analytical percolation concepts can be combined with ANN-based prediction. Sui et al. [33] introduced a graph attention network approach in which the conductive microstructure was represented in a graph-based form, allowing the model to learn from the topology of the conductive network rather than only from global composite descriptors.

Despite their promise, data-driven and machine learning models have important limitations. A major challenge is dataset scarcity. Available datasets are often small, material-specific, and collected using different fillers, polymer matrices, processing routes, measurement methods, and conductivity units or directions [62,63,66,68]. This makes it difficult to train models that are both accurate and broadly transferable. Overfitting is another concern, especially when complex models such as artificial neural networks are trained on limited datasets with many input descriptors [62,63,66]. In such cases, the model may reproduce the training data well but fail to generalize to new filler systems, polymer matrices, processing conditions, or anisotropic conductivity measurements. Model transferability is therefore a central limitation: a model trained mainly on filler content, filler type, and aspect ratio may not predict the effect of processing changes unless descriptors related to mixing history, shear conditions, mold geometry, cooling, orientation, dispersion, agglomeration, and network connectivity are included [31,32,35,68]. Uncertainty quantification is also rarely treated in sufficient detail, even though experimental scatter, inconsistent microstructural characterization, and uncertainty in input descriptors can strongly affect conductivity predictions [66]. Therefore, data-driven models should not be used as purely empirical replacements for physics-based models. Their most useful role is likely in hybrid frameworks that combine physically meaningful descriptors, processing-related variables, simulation-generated microstructures, network-aware representations, and uncertainty-aware validation.

Since anisotropic conductivity is a central focus of this review, Table 2 summarizes how the main model families represent direction-dependent conductivity. The comparison emphasizes the way anisotropy is introduced in each model family, including separate fitted parameters, orientation-averaged homogenization, explicit network topology, or anisotropy-related data descriptors. It also highlights the main inputs required by each approach and the key limitations.

In addition to comparing model assumptions and input requirements, it is important to examine how the individual models reviewed in this work have been evaluated against experimental data. Table 3 summarizes representative validation or comparison studies for the eight conductivity models discussed in this review, with emphasis on the polymer–filler system, composite preparation method, and orientation-related assumptions or findings. For some models, more than one experimental study is included because different studies highlight different aspects of conductivity prediction, including percolation behavior, processing-induced orientation, tunneling effects, conductive-network formation, and data-driven prediction.

## 3. Key Factors Influencing Conductivity

The predictive accuracy of ECPC conductivity models depends on how well they represent key physical and microstructural factors, including filler orientation, aspect ratio, dispersion state, tunneling distance, contact resistance, interphase properties, and processing-induced morphology. These factors can affect both the percolation threshold and the magnitude and directionality of electrical conductivity, helping explain why a model that works well for one composite system may not be reliable for another. Although these factors are discussed separately below for clarity, they are strongly coupled in real processed composites and should not be treated as independent inputs in predictive conductivity models.

### 3.1. Orientation

Filler orientation is one of the most important microstructural factors controlling the electrical conductivity of carbon-filled polymer composites, especially when the conductive phase consists of high-aspect-ratio fillers such as CNTs or carbon fibers. Unlike spherical particles, elongated fillers conduct preferentially along their long axis and form conductive pathways in a direction-dependent manner. Therefore, orientation affects not only the magnitude of conductivity, but also the anisotropy of the composite and the percolation threshold required to form a connected network. From a modeling perspective, this makes orientation a critical link between the real processed microstructure and the predicted effective conductivity. Analytical and micromechanics-based models usually include orientation through an orientation distribution function or averaged orientation descriptors, such as the orientation factor $\left\langle {cos}^{2}\theta \right\rangle$, where θ is the angle between the filler long axis and the direction of electrical conductivity being evaluated [69]. For a three-dimensional randomly oriented filler system, $\langle {cos}^{2}\theta \rangle =1/3$, whereas larger values indicate preferential alignment along the measurement direction. Chanda et al. [69] showed that orientation can strongly influence the conductivity of random and aligned nanocomposites, but the effect is not always monotonic: although alignment can improve conductivity along the preferred direction, excessive alignment may reduce transverse contacts and weaken the three-dimensional conductive network. This point is important for ECPC modeling because it shows that orientation should not be treated simply as a factor that always increases conductivity; instead, its influence depends on the measurement direction, filler concentration, and the degree of network connectivity.

Several analytical studies have therefore attempted to include orientation explicitly in conductivity models rather than treating it only as a qualitative microstructural feature. Chanda et al. [69] developed analytical models for random and aligned CNT/polymer nanocomposites in which orientation was introduced through the average orientation factor, ⟨${cos}^{2}\theta$⟩, together with filler geometry, interphase, percolation threshold, and tunneling distance. Their work is useful for conductivity modeling because it shows how alignment changes both the effective conductive path length and the percolation behavior of rod-like fillers. For random composites, ⟨${cos}^{2}\theta$⟩ is commonly taken as 1/3, while experimentally aligned systems may show larger values because most fillers are oriented closer to the measurement direction. More recently, Ahmadi and Saxena [30] used an Eshelby-based analytical framework to examine the effect of CNT alignment on longitudinal and transverse conductivity by controlling the filler orientation through a limit angle and probability distribution functions. Their results showed that increasing alignment can enhance conductivity along the preferred direction, but it also strongly reduces transverse conductivity. These studies demonstrate that orientation should be represented as a directional and statistical variable in conductivity models, rather than as a simple aligned/random classification.

An important point that emerges from numerical, analytical, and experimental studies is that the effect of orientation on conductivity is direction-dependent and not always monotonic. Although alignment can increase conductivity along the preferred direction, perfect alignment may reduce the probability of filler–filler contacts in the transverse direction and therefore weaken the three-dimensional conductive network. Bao et al. [73] demonstrated this behavior using a three-dimensional Monte Carlo model with intrinsic and contact resistances, showing that CNT/polymer composites can reach maximum conductivity at a partially aligned state rather than under perfect alignment. This result is consistent with shear-flow simulations by Eken et al. [74], who showed that processing-induced shear changes CNT orientation, agglomerate size, and conductivity by either forming or disrupting conductive aggregates. Experimental evidence for the directional nature of this effect was also reported by Qu et al. [75], who studied aligned carbon-fiber/PMMA composites and showed that conductivity increases when the fibers are oriented along the measurement direction, while the transverse response remains more limited. Similarly, Ahmadi and Saxena [30] showed analytically that increasing CNT alignment strongly decreases transverse conductivity while the longitudinal conductivity is less sensitive to changes in orientation. Together, these studies indicate that orientation should be interpreted as a balance between directional transport and network connectivity, rather than as a simple factor that always improves conductivity.

In practical composite processing, filler orientation is not an independent microstructural variable, but develops together with shear history, dispersion, agglomeration, crystallization, and spatial non-uniformity. This is particularly important for injection-molded and microinjection-molded CNT/polymer composites, where strong shear fields tend to align nanotubes along the flow direction and can therefore change both the percolation threshold and the anisotropic conductivity. Mahmoodi et al. [35] showed for injection-molded MWCNT/PS composites that mold design and processing conditions strongly affect electrical resistivity, confirming that flow-induced CNT orientation influences the formation of conductive pathways. Mi et al. [31] similarly reported for PP/CNT composites that processing routes such as compression molding, conventional injection molding, and interval injection molding produce different orientation and dispersion states, leading to different percolation thresholds and conductivity anisotropy. This processing dependence is also supported by Zhou et al. [32], who found that PP/MWCNT microparts required higher CNT concentrations to form conductive networks than compression-molded samples, and that CNT distribution was non-uniform along the flow direction. Their results further show that the high shear and cooling conditions of microinjection molding can favor CNT alignment along the flow direction but make the construction of a three-dimensional conductive network more difficult at low filler contents. Therefore, conductivity models intended for processed ECPCs should not treat orientation only as an idealized random or aligned state; instead, they should account for spatially varying orientation, processing-induced anisotropy, and the coupling between orientation and network formation.

Overall, orientation should be treated as a directional descriptor rather than a simple aligned/random classification. Its effect on conductivity depends on the measurement direction and on whether alignment promotes or disrupts conductive-network connectivity. This dependence is especially important for high-aspect-ratio fillers, where orientation interacts strongly with filler geometry, aspect ratio, and contact probability; therefore, these geometrical factors are discussed next.

### 3.2. Aspect Ratio and Filler Geometry

Aspect ratio and filler geometry are among the most important morphological descriptors used in modeling the electrical conductivity of carbon-filled polymer composites, because they strongly affect filler–filler interaction probability, tunneling-path formation, and the percolation threshold [76,77,78]. For one-dimensional fillers such as CNTs and carbon fibers, aspect ratio is commonly defined as the ratio of filler length to diameter, while for two-dimensional fillers such as graphene, geometry is more appropriately described by lateral size, thickness, and shape anisotropy [13,76]. This distinction is important because different filler geometries generate different excluded volumes and therefore different probabilities of forming a connected conductive network [13,77]. In continuum percolation and excluded-volume models, high-aspect-ratio rod-like fillers are expected to percolate at lower filler concentrations because each particle occupies a larger interaction volume and has a greater probability of contacting or approaching neighboring fillers [77,78]. This explains why CNTs and carbon fibers can often produce lower percolation thresholds than more compact fillers such as carbon black, provided that their effective aspect ratio is preserved during processing [79]. However, the influence of aspect ratio is not determined only by the nominal length-to-diameter ratio measured before processing; it also depends on filler waviness, dispersion state, alignment, agglomeration, and possible breakage during mixing or molding [60,76,79]. Therefore, conductivity models that include aspect ratio only as an ideal straight-particle parameter may overestimate network formation when real fillers are curved, shortened, bundled, or strongly aligned [60,76,78].

The reduction in percolation threshold with increasing aspect ratio is commonly explained using excluded-volume and connectedness-percolation arguments [77,78,80]. In these approaches, percolation occurs when the average number of geometrical or electrical connections per filler becomes large enough to create a system-spanning cluster [76,77]. For rod-like fillers, increasing the length-to-diameter ratio increases the volume around each filler within which another filler can intersect, contact, or fall within tunneling distance [76,77,78]. As a result, fewer particles are required to form a connected conductive network, and the critical filler volume fraction decreases as aspect ratio increases [76,77,78,80]. This trend has been observed in both continuum percolation simulations and conductivity models for CNT/polymer systems, where high-aspect-ratio fillers show lower percolation thresholds than short rods or compact particles [60,76,77,80]. However, this relationship should not be treated as a universal inverse-aspect-ratio rule, because finite rod length, hard-core interactions, tunneling distance, and particle orientation can cause significant deviations from ideal slender-rod predictions [60,77,78]. Therefore, aspect ratio is best understood as a dominant but not independent parameter: it controls the geometrical opportunity for network formation, but its quantitative effect depends on how connectivity is defined in the model, whether by direct contact, excluded volume, or tunneling distance [60,76,77,80].

Although high aspect ratio is generally favorable for reducing the percolation threshold, the effective aspect ratio in real composites can be substantially lower than the nominal value because carbon nanofillers are rarely present as perfectly straight and individually dispersed particles. CNTs and carbon nanofibers can become curved, wavy, entangled, bundled, or shortened during processing, which reduces their ability to form long-range conductive pathways [76,79]. Yu et al. showed that filler waviness increases the percolation threshold even when the nominal aspect ratio is unchanged, because curved fillers require a larger average number of contacts per filler to form a spanning network [76]. This means that the same nominal aspect ratio can lead to different percolation behavior depending on the actual filler conformation in the polymer matrix [76]. Processing can further modify this effect. Tsuchiya et al. reported that maintaining CNT length during mixing helped preserve the effective aspect ratio and produced a much lower percolation threshold, whereas stronger mechanical shear caused CNT breakage and reduced electrical performance [79]. Therefore, models based on straight rods or ideal cylinders should be interpreted carefully, because they may underestimate the percolation threshold when real fillers are shortened, wavy, or aggregated [76,79].

In addition to lowering the percolation threshold, aspect ratio can also influence the magnitude of conductivity after a connected network has formed. In conductive polymer composites, the total resistance of a current path is controlled not only by the intrinsic resistance of the fillers, but also by the number and resistance of filler–filler junctions, including direct contacts and tunneling gaps [39,60]. Longer fillers can span larger distances within the matrix and may therefore reduce the number of junctions required to form a continuous conductive pathway [39]. This is important because junctions often contribute a large fraction of the total network resistance, especially in CNT/polymer composites where electron tunneling across thin polymer barriers can dominate charge transport [60]. Fang et al. showed that increasing CNT aspect ratio can enhance conductivity after percolation because longer CNTs reduce the number of junctions along conductive paths and improve network efficiency [39]. Similarly, Lu et al. reported that CNT aspect ratio mainly controls the percolation threshold, while tunneling-related parameters such as barrier height strongly affect the maximum conductivity level [60]. Therefore, aspect ratio should be considered together with junction resistance and tunneling distance when evaluating conductivity above the percolation threshold, rather than being treated only as a parameter controlling the onset of percolation [39,60].

From a modeling perspective, the main challenge is that aspect ratio is often introduced as a single idealized geometrical parameter, while real composites contain distributions of filler length, diameter, curvature, orientation, and aggregation state [76,79,81]. In addition to length polydispersity, diameter variation can also affect the effective aspect-ratio distribution, especially for CNTs where diameter may differ between single-walled, few-walled, and multi-walled structures or between different commercial grades [3,6,18]. Since aspect ratio is defined by both length and diameter, diameter variation can change the excluded volume, contact probability, tunneling-gap distribution, and local resistance of the conductive network. Therefore, conductivity models that use only a single nominal diameter may oversimplify the real percolation behavior of CNT-filled composites. This simplification can be useful for analytical models and parametric studies, but it may limit quantitative accuracy when processing changes the effective filler geometry or when conductivity is controlled by a small number of critical contacts [76,79]. Majidian et al. showed that CNT length polydispersity and clustering can substantially affect the electrical conductivity of CNT/polymer composites, confirming that a single nominal aspect ratio may be insufficient to describe real conductive networks [81]. Numerical models such as Monte Carlo, finite-element, and resistor-network approaches can represent these effects more explicitly by assigning filler dimensions, orientations, tunneling distances, and junction resistances at the microstructural level [39,60]. However, these models also require more detailed data and higher computational cost than analytical percolation or homogenization approaches [39,60]. Therefore, aspect ratio should be treated not only as a material property of the filler, but also as a microstructural descriptor that depends on dispersion quality, processing conditions, particle-size distribution, and the way electrical connectivity is defined in the model [60,76,79,81].

### 3.3. Dispersion State and Agglomeration

After filler orientation and aspect ratio, the dispersion state and spatial distribution of conductive fillers are among the most important factors controlling the electrical conductivity of carbon-filled polymer composites. Even when the filler content, intrinsic conductivity, and nominal aspect ratio are fixed, the final conductivity can change significantly depending on whether the fillers are uniformly dispersed, partially clustered, severely agglomerated, or arranged in a segregated network. This is especially important for CNT- and graphene-based composites, because their high surface area, high aspect ratio, and strong van der Waals interactions make them prone to aggregation in polymer matrices [8,9,82]. Poor dispersion can prevent the formation of an effective conductive network, while excessive separation of well-dispersed fillers may also reduce filler–filler contacts and increase the average tunneling distance. Therefore, the effect of dispersion on conductivity is not always monotonic; rather, the key requirement is to achieve a suitable balance between filler dispersion and conductive-network formation [83].

Pan et al. [83] showed that improved interfacial adhesion and dispersion of MWCNTs in polypropylene could reduce electrical conductivity when the formation of CNT–CNT connections was hindered, whereas post-heat treatment increased conductivity by promoting network formation. Similarly, Mutiso and Winey [3] emphasized that filler distribution within the polymer matrix is one of the dominant factors determining both the percolation threshold and the composite conductivity, and that heterogeneous or segregated filler arrangements can sometimes produce efficient conductive networks at very low filler loadings.

From a modeling perspective, dispersion and agglomeration are challenging to incorporate because they cannot usually be represented by a single material parameter. Unlike filler volume fraction or nominal aspect ratio, dispersion describes the spatial arrangement of fillers, including local filler concentration, cluster size, interparticle-distance distribution, and connectivity between filler-rich regions. Many analytical and micromechanics-based models assume that fillers are uniformly and randomly distributed in the matrix, which simplifies the problem but may not represent real carbon-filled polymer composites. This simplification can be particularly important near the percolation threshold, where conductivity is controlled by a small number of critical connections and the local arrangement of fillers can dominate the macroscopic response. Tang et al. [82] noted that previous analytical models often ignored the combined effects of CNT waviness and dispersion, although these features inevitably exist in real CNT/polymer composites and significantly affect both the percolation threshold and electrical conductivity. Mora et al. [84] also discussed that agglomeration and segregation can strongly modify the conductive network in CNT/polymer composites, making the spatial distribution of CNTs an essential factor for conductivity modeling. Therefore, models that rely only on average filler content and idealized random distributions may fail to capture the real network topology formed by clustered, bundled, or non-uniformly distributed fillers.

Several experimental studies confirm that the relationship between dispersion and electrical conductivity is not straightforward. In many cases, improving the dispersion of CNTs helps distribute the conductive phase more uniformly through the polymer matrix, but this does not necessarily lead to higher conductivity if the nanotubes become too isolated to form continuous conductive paths. Pan et al. [83] studied MWCNT/PP composites prepared by melt blending and showed that chemical modification and compatibilization could improve the dispersion of MWCNTs, but the improved interfacial adhesion between CNTs and the polymer could also reduce conductivity by limiting CNT–CNT network formation. They further reported that post-heat treatment increased the conductivity of several samples by several orders of magnitude because CNT connections were re-established after processing. This indicates that conductivity depends not only on how uniformly the fillers are dispersed, but also on whether the dispersion state allows the formation of continuous conductive pathways. A similar conclusion was reached by Mi et al. [31], who showed that different molding routes produced different CNT dispersion and orientation states in PP/CNT composites, leading to different percolation thresholds. In their study, high-shear interval injection molding increased CNT agglomerate dispersion and orientation, but it could also destroy an already formed conductive network and increase the percolation threshold. These results show that an optimum or moderate dispersion state may be more favorable than either severe agglomeration or complete separation of fillers, because electrical conductivity requires both sufficient filler distribution and sufficient filler–filler connectivity.

Several modeling strategies have been proposed to represent dispersion and agglomeration at different levels of detail. Simpler analytical models usually represent these effects through average descriptors, such as a dispersion index, an agglomeration parameter, an effective filler concentration, or the fraction of fillers that belong to the conductive network. For example, Tang et al. [82] introduced a dispersion index and a percolated CNT fraction to account for the effects of CNT distribution and network formation on conductivity, showing that models assuming uniform dispersion can overpredict conductivity when real CNT agglomeration is present. More explicit approaches, such as Monte Carlo simulations and resistor-network models, can represent filler positions, clusters, tunneling distances, and conductive junctions more directly, but they require detailed microstructural inputs and higher computational cost. This limitation is consistent with the broader discussion by Mutiso and Winey [3], who emphasized that dispersion is difficult to quantify and that no single descriptor fully captures filler distribution across different length scales. Therefore, although dispersion can be incorporated into conductivity models, it remains one of the most difficult microstructural factors to represent accurately, especially when filler clustering, tunneling, orientation, and processing-induced morphology occur simultaneously.

### 3.4. Coupled Effects of Orientation, Aspect Ratio, and Dispersion

Although orientation, aspect ratio, and dispersion are often introduced as separate descriptors in conductivity models, their effects on electrical conductivity are strongly coupled in real carbon-filled polymer composites. For high-aspect-ratio fillers such as CNTs and carbon fibers, the nominal aspect ratio affects the probability of contact or tunneling between neighboring fillers. However, the effective aspect ratio within the composite can be reduced by waviness, breakage, bundling, or agglomeration during processing [76,79,81]. Filler orientation then determines how this effective aspect ratio contributes to network formation in different directions. Alignment can increase conductivity along the preferred direction, but excessive alignment may reduce transverse contacts and weaken three-dimensional conductive-network connectivity [30,73,74,75].

Dispersion and agglomeration further modify this relationship. Improved dispersion can distribute fillers more uniformly and reduce large agglomerates, but it does not necessarily increase conductivity if the fillers become too isolated to form continuous conductive pathways. Conversely, moderate clustering or segregated filler arrangements may promote conductive-network formation at lower filler contents, whereas severe agglomeration can reduce the effective network volume and increase local heterogeneity [3,82,83,84]. Therefore, the same filler content and nominal aspect ratio can produce different conductivity values depending on how processing changes orientation, dispersion state, interparticle spacing, and the number of effective filler–filler contacts.

This coupling is especially important in processed ECPCs, where mixing, shear history, mold geometry, cooling, and post-processing can simultaneously affect filler alignment, breakage, dispersion, agglomeration, and tunneling distance [31,32,35]. Therefore, treating orientation, aspect ratio, and dispersion as independent scalar inputs can limit the reliability of anisotropic conductivity prediction, particularly when processing produces spatially varying microstructures. More realistic predictive frameworks should represent these descriptors jointly, for example, by combining orientation distributions, effective aspect-ratio distributions, dispersion or agglomeration descriptors, and network-level measures such as contact density, tunneling-distance distribution, and anisotropic connectivity.

### 3.5. Interfacial Effects and Tunneling Distance

Interfacial effects play an important role in the electrical conductivity of carbon-filled polymer composites because charge transport is strongly influenced not only by the intrinsic conductivity and concentration of the filler, but also by the interaction between the filler surface and the surrounding polymer matrix. The surface properties of both phases can affect polymer wetting, filler dispersion, polymer coating around the filler, and the probability of filler–filler contact or near-contact. Mamunya et al. [45] emphasized that polymer–filler interactions can significantly influence the conductivity of filled polymers and incorporated interfacial effects through surface-energy-related parameters in their conductivity model. Clingerman et al. [26] later showed that models including both filler geometry and surface-energy effects could better describe the conductivity of several conductive polymer composites than simpler models that considered only filler volume fraction. More recently, Alayli et al. [85] provided further evidence that surface energy can strongly affect the electrical conductivity of polymer-matrix composites, showing that the same conductive filler may produce different conductivity levels depending on the polymer matrix. These studies indicate that interfacial effects should not be treated only as secondary material details; rather, they can directly influence conductive-network formation by changing filler dispersion, contact quality, and the thickness of polymer-rich regions between neighboring conductive particles.

In addition to direct filler–filler contact, electron tunneling through thin polymer gaps is another important mechanism controlling electrical conductivity in carbon-filled polymer composites. This means that two conductive particles may contribute to the same electrical network even when they are not physically touching, provided that the separation distance is small enough for electron transport across the insulating matrix. Balberg [86] discussed this distinction in carbon-black/polymer composites and showed that conductivity cannot always be interpreted using geometrical percolation alone, because tunneling resistance between neighboring particles can strongly affect the observed electrical behavior. Li et al. [87] also used an interparticle-distance criterion to estimate the percolation threshold of graphite-nanoplatelet/polymer composites, showing that the critical separation distance between conductive fillers can directly influence the onset of electrical connectivity. Therefore, tunneling distance acts as a bridge between filler morphology and network formation: a larger allowable tunneling distance increases the probability of electrical connection between particles, while a larger polymer gap increases tunneling resistance and reduces the effectiveness of conductive pathways.

In conductivity models, tunneling is commonly represented by assigning electrical resistance to the thin polymer gap between neighboring conductive fillers. This resistance usually increases rapidly as the separation distance increases, which means that small changes in the interparticle distance can lead to large changes in the predicted conductivity. Hu et al. [40] incorporated tunneling resistance into a CNT/polymer nanocomposite network model and showed that the electrical response is strongly affected by the resistance assigned to CNT–CNT junctions separated by thin polymer gaps. Mohiuddin and Hoa [88] also emphasized the importance of junction resistance in CNT/polymer composites, distinguishing the effect of CNT–CNT contact resistance from the intrinsic resistance of the nanotubes themselves. These studies show that modeling ECPC conductivity requires more than identifying whether fillers are connected or disconnected; the quality of each electrical connection must also be represented. Therefore, tunneling distance is usually used as either a connectivity criterion, defining whether two fillers are electrically connected, or as an input to a tunneling-resistance expression that determines the resistance of each filler–filler junction.

More recent models have tried to represent interfacial and tunneling effects more explicitly by separating the composite into filler, interphase, tunneling, and matrix regions. Razavi et al. [89] modeled CNT/polymer nanocomposites by considering conductive interphase regions around nanotubes and tunneling regions between neighboring conductive phases. Liu et al. [90] similarly treated the nanoparticle, interphase, and tunneling regions as distinct contributors to the overall conductivity, allowing the effects of their volume fractions and intrinsic resistances to be included. These approaches are useful because they recognize that the polymer region near the filler surface may not behave exactly like the bulk matrix and that electron transport may occur through nanoscale gaps before direct physical contact is established. However, they also require additional parameters, such as interphase thickness, interphase conductivity, tunneling distance, and tunneling resistance, which are difficult to measure directly and are often estimated or fitted. Therefore, interfacial effects and tunneling distance are essential for realistic conductivity modeling, but they also introduce uncertainty because small changes in nanoscale interparticle separation or assumed interphase properties can strongly affect the predicted conductivity.

## 4. Current Gaps and Recommendations

Although several modeling approaches have been developed to predict the electrical conductivity of carbon-filled polymer composites, no single model is suitable for all filler types, concentration ranges, processing conditions, and conductivity directions. Model selection depends on the prediction objective and on the dominant transport mechanism in the composite. Percolation-centered models are useful for describing the onset of conductivity and the sharp increase near the percolation threshold, but they are less reliable outside this narrow concentration region and often rely on fitted or semi-empirical parameters. Homogenization models are efficient for estimating averaged effective conductivity when the microstructure can be represented by simplified shape, volume-fraction, and orientation descriptors, but they cannot directly resolve conductive-network formation, tunneling paths, or local filler–filler contacts. Network-based models provide a more explicit representation of connectivity and resistance pathways, but their accuracy depends strongly on the quality of the microstructural inputs and the assumed contact or tunneling parameters. Data-driven models can include many formulation, processing, and microstructural variables, but their reliability depends on the size, quality, and representativeness of the training data. Recent attempts to combine homogenization, percolation, and equivalent-circuit concepts further confirm that integrated frameworks are increasingly needed to describe conductivity over a wider range of filler concentrations and composite morphologies [42].

A major remaining challenge is the limited ability of current models to represent real processing-induced microstructures. Many models still assume idealized random, uniform, or globally aligned filler distributions, whereas processed composites often contain spatially varying orientation, non-uniform dispersion, agglomeration, and layered structures. These features are especially important for high-aspect-ratio fillers such as CNTs and carbon fibers, because orientation can enhance conductivity in one direction while reducing transverse connectivity and increasing anisotropy [30].

Another important gap is the simplified treatment of filler geometry, dispersion, and interfacial transport. Many models use nominal aspect ratio or average filler content, although real fillers may become shortened, curved, bundled, or clustered during processing. In addition, tunneling distance, contact resistance, interphase thickness, and barrier height are difficult to measure directly, even though small changes in these parameters can strongly affect the predicted conductivity [91]. Hybrid filler systems add further complexity because fillers with different geometries and conductivities may form multiscale networks that are not fully captured by single-filler models. Recent studies on CNT/CB and graphene/CNF hybrid systems show progress in this direction, but they also confirm that mixed-dimensional and synergistic conductive networks remain difficult to predict [24,92].

Future research should therefore focus on unified predictive frameworks that connect processing, microstructure evolution, physics-based conductivity modeling, and data-driven prediction. A practical roadmap can be organized into four linked steps. First, processing simulations or experimental characterization should be used to estimate spatially varying microstructural descriptors, such as filler orientation, local concentration, dispersion state, agglomeration, and effective aspect-ratio distribution. Second, these descriptors should be transferred into physics-based conductivity models, including orientation-sensitive homogenization models for averaged direction-dependent properties and resistor-network models for local connectivity, tunneling distance, and current-carrying network topology. Third, data-driven methods should be used to accelerate prediction, identify dominant descriptors, and calibrate uncertain parameters such as tunneling distance, contact resistance, and network-connectivity criteria. Finally, the integrated framework should be validated against anisotropic conductivity measurements from processed composites. Such a roadmap would move conductivity prediction from isolated empirical fitting toward processing-aware, microstructure-informed, and uncertainty-aware modeling of anisotropic ECPCs.

For anisotropic conductivity prediction, the most useful modeling approaches are those that can distinguish between measurement directions and can incorporate orientation-sensitive microstructural descriptors. Simple percolation power-law and scalar effective-medium models are useful for identifying the onset of conductivity, but they are generally insufficient for anisotropic systems because they do not distinguish longitudinal, transverse, and through-thickness transport. Fiber-contact and modified GEM-type models can include orientation or separate in-plane and through-plane conductivity, but they remain semi-empirical and require fitted parameters. Mori–Tanaka and Eshelby-based homogenization models are more suitable when the filler orientation distribution is known, because they can use inclusion shape, anisotropic filler conductivity, and orientation averaging to estimate direction-dependent effective conductivity. However, they do not directly resolve filler–filler contacts or tunneling pathways. Network-based and resistor-network models are therefore especially valuable for anisotropic ECPCs because they can explicitly represent filler positions, orientations, contact probability, tunneling distance, and current-carrying paths in different directions. A practical future direction is to combine processing simulations, which provide spatially varying orientation and concentration fields, with orientation-sensitive homogenization or resistor-network models to predict local and overall anisotropic conductivity.

## Figures and Tables

**Table 1:** Representative morphology and electrical conductivity of common carbon fillers relevant to ECPC conductivity modeling.

Carbon Filler	Geometry	Aspect Ratio	Electrical Conductivity (S/m)
Carbon black (CB)	Aggregated quasi-spherical particles [14,15]	Diameter ≈ 10–50 nm; aggregate-forming [14,15]	~$10^{2}$ S/m for compacted powder [16]
Graphite	Layered platelets/flakes [16]	Micron-scale flakes/platelets; lateral size commonly ~1–300 µm depending on grade [16]	~$10^{3}$ S/m for compacted powder [16]
Graphene/GNP	2D nanosheets/nanoplatelets	Lateral size: ~0.5–10 µm; thickness: ~1.6–60 nm [17]	~$10^{2}$ S/m for compacted powder [16]
SWCNT	1D nanotubes	Diameter: ~1–2 nm; length: µm-scale; AR ~$10^{2}–10^{4}$ [3,6,18]	~$10^{6}$ S/m [18]
MWCNT	1D multi-shell nanotubes	Diameter: ~5–100 nm; length: µm-scale; AR ~$10^{2}–10^{4}$ [3,6]	~$10^{2}$ S/m [16]
Carbon fiber (CF)	1D microfibers	Diameter: ~5–10 µm; length depends on chopped/continuous form [19,20]	~$10^{6}$ S/m [19,20]

**Table 2:** Comparative summary of reviewed conductivity-model families.

Model Family Reviewed	Examples Discussed	Main Representation of Conductivity	Key Inputs	Ability to Represent Anisotropy	Main Limitation
Percolation-centered models	Power-law model; Mamunya model	Conductivity is described through the onset of a connected conductive network above percolation threshold.	Filler volume fraction, percolation threshold, critical exponent, pre-exponential factor, and, for some models, surface-energy or interfacial parameters.	Limited. Directional conductivity can be compared only by fitting separate parameters for different measurement directions.	The fitted parameters do not explicitly describe filler orientation, tunneling distance, dispersion, or conductive-network topology.
Homogenization models	Maxwell-Garnett; Bruggeman; Eshelby; Mori-Tanaka	The heterogeneous composite is replaced by an equivalent homogeneous medium using averaged constituent properties and simplified morphological descriptors.	Matrix and filler conductivity, filler volume fraction, particle shape/aspect ratio, and, in advanced forms, interphase properties and orientation distributions.	Moderate. Eshelby- and Mori–Tanaka-type models can include orientation distributions and predict direction-dependent effective conductivity.	Local filler–filler contacts, tunneling paths, agglomeration, and real conductive-network topology are not explicitly resolved.
Network-based models	Monte Carlo-generated RVE and resistor network models	Conductive fillers and filler–filler junctions are represented as an electrical network with intrinsic, contact, and tunneling resistances.	Filler geometry, position, orientation, aspect ratio, tunneling cutoff distance, contact/tunneling resistance, RVE size, and boundary conditions.	High. Conductivity can be calculated in different directions by applying electrical loading along different axes.	Predictions depend strongly on realistic microstructural inputs and uncertain resistance parameters, and solving large RVEs with many fillers and junctions can be computationally expensive.
Data-driven and machine learning models	Artificial neural networks; interpretable machine learning; graph-based models	Conductivity is learned from experimental, numerical, or microstructural datasets rather than predicted from a closed-form equation.	Training data, filler type/content, material descriptors, processing variables, orientation descriptors, morphology descriptors, and/or network descriptors.	Potentially high, but only when anisotropic conductivity data or orientation/network descriptors are included.	Transferability, interpretability, dataset quality, and uncertainty remain major limitations.

**Table 3:** Representative experimental validation or comparison studies for the conductivity models reviewed in this work.

Model	Validation or Comparison Study	Polymer and Filler Used in the Experiment	Composite Preparation Method	Orientation Assumption or Key Finding
Classical power-law percolation model	Mi et al. [31]	Polypropylene (PP)/carbon nanotubes (CNTs)	Compression molding, conventional injection molding, and interval injection molding	Power-law fitting was used to estimate percolation behavior; increased CNT alignment directed current along the orientation direction, while high agglomerate dispersion could weaken conductive-network formation.
	Chanda et al. [69]	Epoxy resin/graphitized vapor-grown carbon nanofiber (CNF)	Ultrasonic dispersion of CNF in epoxy resin, followed by curing; electric-field alignment was used for aligned samples	Random and electric-field-aligned CNF/epoxy samples were validated separately; orientation factor and percolation threshold were used to capture alignment-dependent conductivity.
Mamunya semi–empirical model	Clingerman et al. [26]	Polypropylene (PP)/carbon black, synthetic graphite, and milled pitch-based carbon fibers	Extrusion followed by injection molding	Mamunya-type prediction included filler aspect ratio and polymer–filler surface energy; orientation was characterized experimentally but not used to predict direction-dependent conductivity.
Maxwell–Garnett effective-medium model	Clingerman et al. [26]	Polypropylene (PP)/carbon black, synthetic graphite, and milled pitch-based carbon fibers	Extrusion followed by injection molding	Maxwell–Garnett-type prediction was evaluated as a scalar effective-medium model; filler orientation was characterized experimentally but not used to predict direction-dependent conductivity.
Bruggeman effective-medium model	Clingerman et al. [26]	Polypropylene (PP)/carbon black, synthetic graphite, and milled pitch-based carbon fibers	Extrusion followed by injection molding	Bruggeman-type prediction was evaluated as a scalar effective-medium model; processing-induced filler orientation was not used to predict direction-dependent conductivity.
Eshelby equivalent inclusion method	Ahmadi and Saxena [30], using Wang et al. [70] experimental data	Polystyrene (PS)/multi-walled carbon nanotubes (MWCNTs)	PS/MWCNT nanocomposite foams prepared by freeze-drying; THF was used as the PS solvent and sonication was used to disperse CNTs	Experimental validation assumed uniform CNT distribution; after validation, the PS/MWCNT case was used to study how CNT orientation changes longitudinal and transverse conductivity.
	Ahmadi and Saxena [30], using Kim et al. [71] experimental data	Epoxy resin/chemically modified MWCNTs	Chemical oxidation of MWCNTs, followed by dispersion in epoxy and curing	Experimental validation used isotropic conductivity data; the model later showed that CNT alignment mainly reduces transverse conductivity while longitudinal conductivity is less sensitive.
Mori–Tanaka micromechanics model	Feng and Jiang [21], using Gojny et al. [72] experimental data	Modified DGEBA-based epoxy resin/SWCNT	Three-roll milling of SWCNTs in epoxy, mixing before hardening, followed by curing for 48 h	CNTs were assumed uniformly dispersed; electron hopping and conductive-network contribution were needed to reproduce the SWCNT/epoxy conductivity trend.
	Feng and Jiang [21], using Kim et al. [71] experimental data	Epoxy resin/chemically modified MWCNT	Acetone/surfactant treatment and sonication, followed by two-roll milling with epoxy and curing	CNTs were assumed uniformly dispersed; the model matched the MWCNT/epoxy trend only when conductive-network contribution was included.
Resistor network model/Monte Carlo network model	Chang et al. [38]	Polypropylene (PP)/multi-walled carbon nanotubes (MWCNTs)	Melt mixing followed by physical foaming in an injection-molding process	Filler alignment caused by deformation changed vertical and lateral percolation thresholds differently; the resistor network used intrinsic and tunneling resistances to reproduce the experimental conductivity response.
	Hu et al. [40]	Polymer nanocomposite/multi-walled carbon nanotubes (MWCNTs)	Fabricated MWCNT/polymer nanocomposite strain sensor; electrical resistance measured during tensile strain	Tunneling resistance between neighboring CNTs and CNT reorientation were included; tunneling was identified as the main mechanism controlling piezoresistivity under small strain.
Artificial neural network/data-driven model	Cavalcanti et al. [68]	Bio-based high-density polyethylene (Bio-HDPE)/carbon black (CB), with additional PP/CB, PET/CB, and Nylon/CB literature datasets	Internal mixing followed by compression molding for Bio-HDPE/CB films; additional literature datasets used for ANN training	Orientation was not included; ANN prediction used filler content, mixing rotor speed, CB surface area, and matrix flow rate as input variables.
	Sui et al. [33]	Homopolymer/CNT nanocomposite network structures	Simulated conductive-network datasets generated using hybrid particle-field molecular dynamics; no single experimental preparation route	Conductive-network topology was encoded as graph features; anisotropy would require direction-dependent network descriptors or directional conductivity outputs.

## Data Availability

The original contributions presented in this study are included in the article. Further inquiries can be directed to the corresponding author.

## Abbreviations

The following abbreviations are used in this manuscript:

ANN	Artificial neural network
CB	Carbon black
CNT	Carbon nanotube
ECPC	Electrically conductive polymer composite
EIM	Equivalent inclusion method
EMI	Electromagnetic interference
ESD	Electrostatic dissipation
GNP	Graphene nanoplatelet
MWCNT	Multi-walled carbon nanotube
RNM	Resistor network model
RVE	Representative volume element
SWCNT	Single-walled carbon nanotube
